# Quantitative* In Vitro* and* In Vivo* Evaluation of Intestinal and Blood-Brain Barrier Transport Kinetics of the Plant* N*-Alkylamide Pellitorine

**DOI:** 10.1155/2016/5497402

**Published:** 2016-07-04

**Authors:** Lieselotte Veryser, Nathalie Bracke, Evelien Wynendaele, Tanmayee Joshi, Pratima Tatke, Lien Taevernier, Bart De Spiegeleer

**Affiliations:** ^1^Drug Quality and Registration (DruQuaR) Group, Faculty of Pharmaceutical Sciences, Ghent University, Ottergemsesteenweg 460, 9000 Ghent, Belgium; ^2^CU Shah College of Pharmacy, SNDT Women's University, Santacruz West, Mumbai 400 049, India

## Abstract

*Objective.* To evaluate the gut mucosa and blood-brain barrier (BBB) pharmacokinetic permeability properties of the plant* N*-alkylamide pellitorine.* Methods.* Pure pellitorine and an* Anacyclus pyrethrum *extract were used to investigate the permeation of pellitorine through (1) a Caco-2 cell monolayer, (2) the rat gut after oral administration, and (3) the BBB in mice after intravenous and intracerebroventricular administration. A validated bioanalytical UPLC-MS^2^ method was used to quantify pellitorine.* Results.* Pellitorine was able to cross the Caco-2 cell monolayer from the apical-to-basolateral and from the basolateral-to-apical side with apparent permeability coefficients between 0.6 · 10^−5^ and 4.8 · 10^−5^ cm/h and between 0.3 · 10^−5^ and 5.8 · 10^−5^ cm/h, respectively. In rats, a serum elimination rate constant of 0.3 h^−1^ was obtained. Intravenous injection of pellitorine in mice resulted in a rapid and high permeation of pellitorine through the BBB with a unidirectional influx rate constant of 153 *μ*L/(g·min). In particular, 97% of pellitorine reached the brain tissue, while only 3% remained in the brain capillaries. An efflux transfer constant of 0.05 min^−1^ was obtained.* Conclusion.* Pellitorine shows a good gut permeation and rapidly permeates the BBB once in the blood, indicating a possible role in the treatment of central nervous system diseases.

## 1. Introduction 

Pellitorine (deca-2E,4E-dienoic acid isobutylamide, F3M1 according to the FxMy classification of* N*-alkylamides (NAAs)) is a highly abundant and biologically potent diene NAA found in Asteraceae plants such as* Anacyclus pyrethrum* (AP) [1]. Various pharmacological activities of pellitorine have already been reported, namely, antiprotozoal, larvicide, antiseptic, antithrombotic, antituberculosis, antibacterial, anticancer, and antiplatelet aggregation properties and vascular barrier protective effects [2–10]. It was also demonstrated that pellitorine reduces fatty acid uptake* in vitro* [11]. Central nervous system (CNS) activities of purified pellitorine are not yet described, while a limited number of CNS activities of* Anacyclus pyrethrum *extracts have been reported. Antiseizure activity was demonstrated after intraperitoneally administration of 100–800 mg/kg of the chloroform fraction of* Anacyclus pyrethrum* roots to mice. Other studies with the* Anacyclus pyrethrum* extract in mice showed anticonvulsant and myorelaxation activities [12]. Another study with an ethanolic AP root extract showed anticonvulsant effect against maximal electro shock (MES) induced convulsions in mice [13]. Sujith et al. [14] found that the AP root extract possesses antidepressant activity in albino Wister rats and improves the learning acquisition of rats.

CNS effects of compounds require them to penetrate the blood-brain barrier in order to reach their brain target. Compounds can enter the systemic circulation after topical and oral administration, by crossing several physiological barriers (i.e., stratum corneum, intestinal barrier), and further reach the brain. A previous study of our research group demonstrated that pellitorine permeates human skin using an* in vitro* Franz diffusion cell (FDC) experiment. The aqueous permeability coefficient *K*_*p*,*aq*_ of pellitorine was 2.3 · 10^−4^ cm/h for the crude plant extract and 1.1 · 10^−4^ cm/h for purified pellitorine [15]. Moreover, the BBB transport kinetics of spilanthol were studied as well [16].

Up till now, to our best knowledge, no information is available about the intestinal barrier and blood-brain barrier transport kinetics of the 2,4-diene* N*-alkylamide pellitorine. Hence, the aim of this study was to quantitatively investigate this* in vitro* using a Caco-2 cell monolayer as well as* in vivo* with an oral gavage rat model and a BBB mice model.

## 2. Materials and Methods 

### 2.1. Chemicals and Reagents

Dextran was bought from AppliChem GmbH (Darmstadt, Germany), while trypsin-EDTA came from Invitrogen (Ghent, Belgium). UPLC-MS grade acetonitrile (ACN), methanol (MeOH), and trifluoroacetic acid (TFA) were purchased from Biosolve (Valkenswaard, Netherlands). Dimethylacetamide and phosphoric acid (85%) (H_3_PO_4_) were obtained from Jansen Chimica (Geel, Belgium), while propylene glycol (PG) was bought from Riedel-de Haën (Seelze-Hannover, Germany). Disodium hydrogen phosphate dihydrate (Na_2_HPO_4_·2H_2_O), sodium hydrogen carbonate (NaHCO_3_), sodium dihydrogen phosphate monohydrate (NaH_2_PO_4_·H_2_O), acetic acid, and absolute ethanol (EtOH, ≥99.9% V/V) were purchased from Merck KGaA (Darmstadt, Germany), while polyethylene glycol 400 (PEG 400), calcium dichloride (CaCl_2_), LC-MS grade formic acid (FA), tween 80, sodium hydroxide (NaOH), D-glucose, HEPES, Hanks' Balanced Salt Solution (HBSS), vitamin E-TPGS, trypan blue, dimethylsulfoxide (DMSO), sodium chloride (NaCl), potassium chloride (KCl), phosphate buffered saline (PBS), sodium hydrogen carbonate (NaHCO_3_), calcium chloride dehydrate (CaCl_2_·2H_2_O), sodium lactate, sodium dihydrogen phosphate (NaH_2_PO_4_), sodium sulphate (Na_2_SO_4_), urethane, Krebs-Henseleit buffer, and hydrochloric acid (HCl) were bought from Sigma-Aldrich (Diegem, Belgium). HPLC gradient grade ACN, MeOH, and absolute ethanol (99.8% V/V) were obtained from Fisher Scientific (Erembodegem, Belgium). Ultrapure water (H_2_O) of 18.2 MΩ·cm quality was produced by an Arium 611 purification system (Sartorius, Göttingen, Germany).

### 2.2. Products Examined

The ethanolic* Anacyclus pyrethrum* root extract was prepared and characterised as previously described [17]. The* Anacyclus pyrethrum* root extract (extract contains 4.87% w/w NAAs of which pellitorine was the main NAA (1.55% w/w pellitorine)) was used for the oral gavage experiment and the Caco-2 cell permeability assay. Pellitorine was bought from Adipogen Life Sciences (99.8% purity determined by HPLC) and was used for the BBB transport assay. As analytical internal standard (IS), isobutyldecanamide was used, obtained from the Laboratory of Medicinal Chemistry (Ghent University). Dermorphin (>95% purity determined by HPLC) was obtained from Bachem (Bubendorf, Switzerland) and bovine serum albumin (BSA) from Merck KGaA (Darmstadt, Germany); these compounds were used as positive and negative controls during BBB studies, respectively.

### 2.3. *In Vitro* Permeation Study in Caco-2 Cell Monolayers

#### 2.3.1. Cell Culture

The Caco-2 cell line originated from a human colorectal carcinoma and was maintained in Dulbecco's modified Eagle's medium (DMEM) (95% humidity, 37°C, 5% CO_2_), supplemented with 10% (V/V) fetal bovine serum, 100 U/mL penicillin and 100 *μ*g/mL streptomycin, 2 mM L-glutamine, and 1% nonessential amino acids (all from Invitrogen/GIBCO, Ghent, Belgium).

#### 2.3.2. Caco-2 Cell Permeability Assay

The Caco-2 cell intestinal model was used to investigate the gut mucosa permeation of pellitorine. On each Transwell (Corning Costar, New York, USA) membrane insert filter (0.4 *μ*m pore size, 12 mm filter diameter), the cells were seeded at a density of 2.6 · 10^5^ cells/cm^2^ cells and cultivated in the described supplemented DMEM. Every second day, the medium was changed. During a period of 21–29 days, the cells were grown and differentiated until monolayers were formed. The integrity of the monolayers was checked via the measurement of transepithelial electrical resistance (TEER) of the monolayers with a Millicell-ERS system (Millipore Corp., Bedford, MA, USA) before and after the transport experiments. The experiments were performed in duplicate for each dose solution. Transport experiments were carried out according to Hubatsch et al. [18] in two directions**: **from the apical-to-basolateral (*ab*) direction and from the basolateral-to-apical (*ba*) direction in Hanks' Balanced Salt solution. Two dose solutions of pellitorine (in* Anacyclus pyrethrum* extract) of 1 *μ*g/mL in 0.5% EtOH (further indicated as DS1) and of 56 *μ*g/mL in 0.5% of a mixture of vitamin E-TPGS (33.3%), EtOH (11.1%), PEG 400 (33.3%), and PG (22.2%) (further indicated as DS2) dissolved in HBSS were tested.

Final apical volumes of 0.4 mL and basolateral volumes of 1.2 mL were used for 12 mm filter supports during the transport experiment. After 15, 30, 60, 90, and 120 min, samples were taken from the receiver compartment (basolateral to apical transport: 100 *μ*L, apical-to-basolateral transport: 300 *μ*L) and immediately replaced by fresh HBSS. After 120 min, a sample from the donor compartment was taken as well and a mass balance was constructed for pellitorine, which ranged between 103.6 and 132.4% total recovery. To confirm the validity of the test, atenolol (50 *μ*M) and propranolol (20 *μ*M) were used as the negative (low permeability) and positive (high permeability) control, respectively [19]. Pellitorine was quantified using a ultraperformance liquid chromatography-tandem mass spectrometry (UPLC-MS^2^) method as described below.

The apparent permeability coefficient (*P*_app_ in cm/s) of pellitorine in DS2 was calculated using a sink condition equation, while *P*_app_ of pellitorine in DS1 was calculated using a nonsink equation. From Fick's first law, *J* = −*D* × [*dC*(*x*)]/*dx* (*J* is the flux or transfer rate along the donor-to-receptor side, *D* is the diffusion coefficient, *x* is the distance from the donor compartment, and *C*(*x*) is the concentration in the barrier at the coordinate *x* in the barrier), the following differential equation is derived: *dM*_*r*_(*t*)/*dt* = *P*_app_ × *A* × [*C*_*d*_(*t*) − *C*_*r*_(*t*)], in which *P*_app_ is the apparent permeability coefficient (a product of distribution coefficient with diffusion coefficient divided by the barrier thickness), *M*_*r*_ is the amount of substance in the receiver chamber, *A* is the cross-sectional area of the barrier, *C*_*d*_ is the donor concentration, and *C*_*r*_ is the receiver concentration. This differential equation can be solved to calculate *P*_app_ using different initial conditions depending on sink or nonsink conditions. In case of sink conditions, the following equation was obtained from the amount of compound transported per time *dM*_*r*_(*t*)/*dt* with the receiver concentration set to zero [18, 20]:

(1) $$\begin{array}{c} \frac{M_{r}\left(t\right)}{C_{d,0}}=P_{app}\times A\times t+c, \end{array}$$

where *P*_app_ is the apparent permeability coefficient (cm/s), *A* is the surface area of the filter (1.12 cm^2^), *C*_*d*,0_ is the initial concentration in the donor chamber (*μ*g/mL), *t* is the time (s), and *c* is a constant term which vanishes, since *M*_*r*_ is initially zero at *t* = 0.

The donor concentration at each time point was calculated by taking into account the amount of compound which already permeated through the cell monolayer during that time interval. In this way, the reduction in donor concentration after every sampling was also taken into account. The cumulative fraction transported FA_cum_ (cm) is defined as

(2) FAcum1A×∑k=1iCrtk−f×Crtk−1×VrCdtk−1+Cdtk/2=Papp×ti

in which *V*_*r*_ is the volume of the receiver compartment (mL), *f* is the sample replacement dilution factor (1 − *V*_*s*_/*V*_*r*_ with *V*_*s*_ being the sample volume), *t*_*i*_ (s) is the time at sampling *i*, *t*_*k*_ (s) is the time at sampling *k*, *C*_*r*_(*t*_*k*_) (*μ*g/mL) is the experimentally determined concentration of pellitorine in the receiver compartment at sampling *k*, and *C*_*d*_(*t*_*k*_) (*μ*g/mL) is the experimentally determined concentration of pellitorine in the donor compartment at sampling *k*. *C*_*d*_ is considered as constant and is obtained by taking the average of *C*_*d*_ of the start and the end of the time interval. *P*_app_ was calculated from the slope of the cumulative fraction versus time curve using a linear fit.

In case of nonsink conditions, the differential equation resulted in the following solution [18, 20]:

(3) Crt=MVd+Vr+Cr,t−1×f−MVd+Vr×e−Papp×A×1/Vd+1/Vr×Δt

in which *M* (*μ*g) is the total amount of pellitorine in the system at time *t*, *V*_*d*_ (mL) is the volume of the donor compartment, *C*_*r*,*t*−1_ (*μ*g/mL) is the concentration of pellitorine in the receiver compartment at the previous time point, *t* is the time (s), Δ*t* (s) is the time at time *t* minus the previous time point, and *C*_*r*_(*t*) (*μ*g/mL) is the concentration of pellitorine in the receiver compartment at time *t*. Minimisation of the sum of squared residuals (SSR) was used on the nonlinear curve fitting to obtain the *P*_app_. The uptake and efflux ratio are calculated as *P*_app,*ab*_/*P*_app,*ba*_ and *P*_app,*ba*_/*P*_app,*ab*_, respectively.

### 2.4. *In Vivo* Pharmacokinetic Experiment with Rats

#### 2.4.1. Animals

Male and female Wistar rats weighing approximately 220 g were obtained from Bharat Serum and Vaccines Pvt. Ltd., Thane, India (registration number 103/99/CPCSEA) and were housed at CU Shah College of Pharmacy, SNDT Women's University, Santacruz, Mumbai, India (registration number 39/99/CPCSEA) where the experiments were carried out. Rats of 7-8 weeks of age were fasted overnight and used for the experiments.

#### 2.4.2. Pharmacokinetic Experiment

A pellitorine dose solution was prepared using the* Anacyclus pyrethrum *extract. To the rats, 1.5 mL of a 0.73 mg pellitorine/g dose solution in 10 : 20 : 30 : 40 (w/w/w/w) EtOH : PG : Vit E-TPGS : PEG 400 was administered using a gavage needle (stainless steel, length of 3 inches, and 2.5 mm internal diameter). As blank, a solution containing EtOH, PG, Vit E-TPGS, and PEG without pellitorine was used. Each dose solution was orally administered to three female and three male rats. 1.5 mL blood was collected from the retro orbital vein at 1, 2, 3, 4, 6, and 8 h after administration of the dose solution and was centrifuged for 20 min at room temperature. Thereafter, the serum samples were immediately frozen at −80°C until bioanalysis. The animals were sacrificed at the end of the experiment by CO_2_ inhalation. Using GraphPad software (La Jolla, USA), the elimination rate constant *k*_*e*_ (h^−1^) is calculated using the following equation of a one-compartment model: *C*(*t*) = *C*_0_ × *e* ^−*k*_*e*_×*t*^, in which *C*(*t*) (ng/mL) is the concentration of pellitorine at time *t* (h) and *C*_0_ the concentration of pellitorine at time *t* = 0 (ng/mL). The elimination half-life *t*_1/2,*e*_ (h) is calculated as ln⁡(2)/*k*_*e*_.

### 2.5. *In Vivo* Blood-Brain Barrier Experiment with Mice

#### 2.5.1. Animals

Seven-to-ten-week-old female, Institute for Cancer Research, Caesarean Derived-1 (ICR-CD-1) mice (Harlan Laboratories, Venray, Netherlands) weighing 29–32 g were used for the BBB transport experiments. The experiments were performed according to the Ethical Committee principles of laboratory animal welfare as approved by Ghent University (Faculty of Veterinary Medicine, number EC2014/128).

#### 2.5.2. Blood-to-Brain Transport

An* in vivo* multiple time regression (MTR) analysis was performed in order to determine whether pellitorine could enter the brain. A dose solution of 1.8 mg/mL pure pellitorine dissolved in 6.9% EtOH, 2.5% dimethylacetamide, and 0.5% tween 80 (all w/w) diluted in lactated Ringer's solution containing 1% BSA was prepared. After anesthetizing the mice intraperitoneally with a 40% (w/V) urethane solution (3 g/kg), the jugular internalis vein and carotid artery were isolated and 20 *μ*L of the pellitorine dose solution was injected into the jugular vein. After 1, 3, 5, 10, 12.5, and 15 min after injection (with start and end in duplicate), blood was collected from the carotid artery. Immediately thereafter, the mice were decapitated and the brains were isolated. Serum was obtained by centrifuging the collected blood at 10 000 ×g for 15 min at 21°C. As negative and positive control, I^125^- BSA and dermorphin were used, respectively, to ensure the validity of the experiment [21–23]. The serum concentrations of pellitorine were plotted against the time (expressed in min). The curve was fitted using a first-order kinetic, two-compartment model: *C*(*t*) = *C*_1_ × *e* ^−*α*×*t*^ + *C*_2_ × *e* ^−*β*×*t*^, in which *C* is the concentration of pellitorine in serum at time *t*, *α* and *β* are hybrid constants, dependent on the rate constants *k*_*e*_ (elimination rate constant), *k*_1,2_ (rate constant between central and peripheral tissue compartment), *k*_2,1_ (rate constant between peripheral tissue and central compartment), *C*_1_ is the concentration obtained after extrapolation of the distribution phase *α* to the *y*-axis, and *C*_2_ is the concentration obtained after extrapolation of the elimination phase *β* to the *y*-axis. The ratio of the pellitorine brain and serum concentration (*μ*L/g) was plotted versus the exposure time (*θ*), which is a derived time variable of the Gjedde-Patlak plot, to determine the BBB influx kinetics of pellitorine [24, 25].

The exposure time is computed as *θ* = ∫_0_^*T*^((*C*_*s*_(*t*) · *dt*)/*C*_*s*_(*T*)) and is defined as the integral of the concentration of pellitorine in serum from start (*t* = 0 min) to time *T*, divided by the concentration of pellitorine in serum at time *T*. The integral of the concentration of pellitorine in serum from zero to time *T* is the area under the curve until time *T*.

The brain uptake of pellitorine was fitted using a biphasic model, as elaborated by Wong et al. [26]:

(4) CbrainTCsTK·θ+Vg·1−e−θ·K1−K/Vg+V0≅K=0Vg·1−e−θ·K1/Vg+V0

in which *C*_brain_(*T*) is the concentration of pellitorine in the brain at time *T* (ng/g), *C*_*s*_(*T*) the concentration of pellitorine in serum at time *T* (ng/*μ*L), *K* the net clearance (*μ*L/(g·min)), *V*_*g*_ the tissue brain distribution volume (*μ*L/g), *K*_1_ the unidirectional clearance from pellitorine from blood to brain (i.e., the unidirectional brain transfer coefficient, also referred to as influx rate) (*μ*L/(g·min)), and *V*_0_ the vascular brain distribution volume (*μ*L/g). The vascular distribution volume of the negative control BSA (14.8 *μ*L/g) was used as *V*_0_ to calculate the brain kinetic parameters of pellitorine.

#### 2.5.3. Capillary Depletion

A capillary depletion experiment was performed to distinguish the transport of pellitorine into the brain, represented by the parenchyma, and part of pellitorine which is trapped by the endothelial cells of the brain, represented by the capillaries. A method of Triguero et al. [27] was used, which was modified by Gutierrez et al. [28, 29]. The mice were anesthetized intraperitoneally with 40% (w/V) urethane solution (3 g/kg). Thereafter, 20 *μ*L of the 1.8 mg/mL pellitorine dose solution was injected into the jugular vein. Ten min after injection, blood was collected from the abdominal aorta and serum was obtained by centrifuging the blood at 10 000 ×g during 15 min at 21°C. Then, after removing the skin of the mice's chest, the aorta is clamped, the jugular veins are severed, and the brain is immediately perfused manually with 20 mL of Lactated Ringer's solution. The mice are decapitated, and the brains are isolated, weighed, and put into tubes (LoBind Eppendorf), to which 525 *μ*L ice-cold capillary buffer (10 mM HEPES, 141 mM NaCl, 4 mM KCl, 2.8 mM CaCl_2_, 1 mM MgSO_4_, 1 mM NaH_2_PO_4_, and 10 mM D-glucose adjusted to pH 7.4) was added. The tubes (LoBind Eppendorf) were homogenized. Next, 1000 *μ*L of 26% ice-cold dextran solution in capillary buffer was added and vortexed and the tubes were subsequently centrifuged at 20 000 ×g for 60 min at 4°C. Parenchyma and fat tissue (supernatant) were transferred into separate tubes (LoBind Eppendorf) and weighed. The capillaries (pellet) were kept in the original tube. The sample preparation of the pellet and the supernatant is the same sample preparation method as used for the mice brains and mice serum, respectively, as described in Section 2.6.

The distribution in the brains was calculated as follows:

(5) $$\begin{array}{c} Fraction\left(\;\%\;\right)=\frac{M_{tissue}}{M_{capillaries}+M_{parenchyma}}\times 100 \end{array}$$

in which *M*_tissue_ is the amount of pellitorine in the capillaries and parenchyma, *M*_capillaries_ the amount of pellitorine in the capillaries, and *M*_parenchyma_ the amount of pellitorine in the parenchyma. The capillary depletion experiment was performed in duplicate and mean values are reported.

#### 2.5.4. Brain-to-Blood Transport

To evaluate the efflux of pellitorine out of the brain into the blood, a previously described* in vivo* method was used [29]. Briefly, the ICR-CD-1 mice were anesthetized with 40% (w/V) urethane solution (3 g/kg). Then, the skin of the skull was removed and a hole was made into the lateral ventricle using a 22 G needle marked with tape at 2 mm at the following coordinates: 1 mm lateral and 0.34 mm posterior to the bregma. Using a syringe pump (KDS100, KR analytical, Cheshire, UK), 1 *μ*L of the 1.8 mg/mL pellitorine dose solution as used for the blood-to-brain influx experiment was injected intracerebroventriculary (ICV) at a speed of 360 *μ*L/h for 10 s. The mice were decapitated after 1, 3, 5, 10, 12.5, and 15 min after injection. Just before decapitation, blood was collected from the abdominal aorta and serum was obtained by centrifuging the blood at 10 000 ×g during 15 min at 21°C. Thereafter, brains were collected. The natural logarithm of the pellitorine concentration in brain (ng/g) was plotted versus time. The efflux rate constant *k*_out_ (min^−1^) is obtained from the negative value of the slope of the linear regression, applying first-order kinetics. From *k*_out_, the efflux brain half-life (*t*_1/2,brain_) (min) is calculated as follows: *t*_1/2,brain_ = ln⁡(2)/*k*_out_.

#### 2.5.5. *In Vitro* Metabolic Stability of Pellitorine

The* in vitro* metabolic stability of pellitorine was evaluated in mouse brain and liver homogenates and mouse serum according to previously described protocols [22, 30]. Using the Pierce Modified Lowry Protein Assay method (Thermo Scientific), the protein content of each tissue homogenate was determined to prepare a stock solution containing a 0.6 mg/mL protein concentration in Krebs-Henseleit buffer (pH 7.4). Briefly, 75 *μ*L of a 0.25 mg/mL pellitorine solution dissolved in 2% (V/V) EtOH in Krebs-Henseleit buffer pH 7.4 containing 3% (m/V) BSA was incubated in 375 *μ*L of serum/organ homogenate and 300 *μ*L of Krebs-Henseleit buffer pH 7.4 while shaking at 750 rpm at 37°C. After 0, 5, 7.5, 15, 60, and 120 min, aliquots of 100 *μ*L were taken and transferred into 0.5 mL tubes (LoBind Eppendorf) containing 100 *μ*L of 1% (V/V) TFA in water. Next, the samples were heated at 95°C for 5 min and subsequently cooled for 30 min in ice. Thereafter, the samples were centrifuged (20 000 ×g for 30 min at 5°C) and 25 *μ*L of the clear supernatant was injected and analysed using a HPLC-UV/MS method of 46 min which was previously described [15]. The HPLC-MS analysis was done on a HPLC system which consisted of a Spectra System SN4000 interface, a Spectra System SCM1000 degasser, a Spectra System P1000XR pump, a Spectra System AS3000 autosampler, and a Finnigan LCQ Classic ion trap mass spectrometer in positive ion mode (all from Thermo, San José, CA, USA) equipped with Waters 2487 Dual Absorbance detector and Xcalibur 2.0 software (Thermo) for data acquisition. Control solutions were also prepared but without pellitorine. Furthermore, control reference solutions were prepared as well with a prior heat inactivation or without tissue homogenate.

### 2.6. Bioanalytics

#### 2.6.1. Serum Sample Preparation

To 60 *μ*L of 4% (V/V) aqueous H_3_PO_4_ solution and 30 *μ*L of the IS solution, 60 *μ*L of the serum samples was added to a 0.5 mL tube (LoBind Eppendorf) and vortexed. By means of solid phase extraction using a positive pressure-96 processor (Waters, Zellik, Belgium), interfering compounds were removed. The sorbent in each well of the HLB Oasis® *μ*elution 96-well plate (Waters, Zellik, Belgium) was preconditioned with 200 *μ*L of MeOH and equilibrated using 200 *μ*L of ultrapure water. Thereafter, 100 *μ*L of the previous serum sample solution was loaded, followed by two washing steps using 200 *μ*L of 5% MeOH in H_2_O and 200 *μ*L of 20% MeOH in H_2_O, respectively. Two times 25 *μ*L ACN was used to elute pellitorine. 25 *μ*L of a 80 : 20 (V/V) H_2_O : MeOH solution was added to the eluate and analysed with a UPLC-MS^2^ method, as described further in Section 2.6.3.

The bioanalytical method for the quantification of pellitorine in serum was basically validated using rat serum, based upon the European Medicines Agency (EMA) guideline on bioanalytical method validation (EMEA/CHMP/EWP/192217/2009) [31]. The limit of detection (LoD) (*S*/*N* = 3) and limit of quantification (LoQ) (*S*/*N* = 10) of pellitorine, determined on the reference standard, were calculated as 0.19 ng/mL and 0.62 ng/mL, respectively, which correspond to 0.36 ng/mL and 1.16 ng/mL in serum, respectively. A matrix factor of 0.97 was observed for pellitorine in serum. The quantification of pellitorine in the mouse serum samples was performed using a calibration curve with pellitorine standards in 40 : 33.33 : 26.67 (V/V/V) MeOH : ACN : H_2_O. The quantification of pellitorine in the rat serum samples was performed using a preextracted spiked matrix calibration curve. Linearity was ensured in a working range from 0.62 ng/mL up to 101 ng/mL, corresponding to 1.16 ng/mL to 189 ng/mL in serum (*R* ^2^ = 0.9997).

The accuracy of the references used for the calibration curves with pellitorine standards in 40 : 33.33 : 26.67 (V/V/V) MeOH : ACN : H_2_O, the preextracted spiked matrix samples, and the quality control (QC) conformed to the specifications, namely, <15% of the nominal value and for the lower limit of quantification (LLoQ) < 20% of the nominal value. Precision was expressed as the coefficient of variation (CV). The within-run CV value did not exceed 15% for the preextracted spiked matrix samples. Also the CV for the LLoQ sample did not exceed 20% and thus all conform to the specification limits. The recovery in serum was 90.65% (18.8 ng/mL to 189 ng/mL in serum concentration range), calculated from the slopes of the preextracted and postextracted spiked matrix samples. No significant carry-over was observed (<20% of LLoQ pellitorine and <5% for IS). The selectivity conformed to the specifications (placebo serum < 20% of LLoQ pellitorine and <5% for IS).

#### 2.6.2. Brain Sample Preparation

The weighed mice brains were transferred into test tubes, 1.0 mL of the IS solution in ACN was added, and the brains were crushed. The brains were shaken for 4 h at 110 rpm at room temperature using an Eppendorf centrifuge 5810 (Eppendorf, Rotselaar, Belgium). Thereafter, the test tube was centrifuged at 250 ×g for 5 min and 800 *μ*L of the supernatant was transferred into other tubes (LoBind Eppendorf). Then, these test tubes were centrifuged again at 20 000 ×g for 5 min at room temperature and 750 *μ*L of the supernatant was evaporated to dryness under nitrogen. Pellitorine was redissolved in 175 *μ*L 90 : 10 (V/V) H_2_O : ACN and 160 *μ*L of this solution was loaded on the HLB Oasis *μ*elution 96-well plate. The same washing and elution steps were applied as for the serum samples.

Again, a basic validation of the bioanalytical method for the quantification of pellitorine in brains was performed. The LoD and LoQ of pellitorine were 41.2 pg/g and 135 pg/g in brain, respectively. A matrix factor of 0.98 was observed for pellitorine in mice brains. Quantification of pellitorine in the brain samples was performed using the preextracted spiked matrix calibration curve (*R* ^2^ = 0.9982). Linearity was demonstrated in a working range of 0.69 ng/mL to 100 ng/mL pellitorine, corresponding to 135 pg/g to 19.6 ng/g in brain. The accuracy of the references used for the calibration curves and the QC samples with the preextracted spiked matrix samples conformed to the specification limits (<15% of the nominal value, LLoQ < 20% of the nominal value), except for one QC reference preextracted spiked matrix sample (17% of the nominal value instead of <15%), but was still acceptable for our purposes. The within-run CV value did not exceed 15% for the preextracted spiked samples. Also the LLoQ did not exceed 20% and thus all conform to the limits. The recovery in mouse brains was 115.5% (0.98 to 19.6 ng/g brain concentration range).

#### 2.6.3. UPLC-MS^2^ Method

A UPLC-MS^2^ method using an Acquity UPLC RP C18 column (50 × 2.1 mm, 1.7 *μ*m, Waters, Zellik, Belgium) with a suitable guard column was used for the quantification of pellitorine, developed on an Acquity UPLC chromatograph coupled to a Xevo*™* TQ-S mass spectrometer (MS) (Waters, Zellik, Belgium) equipped with a triple quadrupole mass analyser and an electrospray ionisation (ESI) source. The column temperature was kept at 30°C, while the sample compartment remained constant at 5°C. The injection volume was 2 *μ*L and the flow rate was set to 0.5 mL/min. A mobile phase was applied consisting of solvent A (0.1% FA in 30 : 70 (V/V) H_2_O : MeOH) and solvent B (0.1% FA in MeOH) in gradient mode as follows: 0–1.6 min 100 : 0 (V/V) A : B, 1.6–2 min going from 100 : 0 (V/V) A : B to 0 : 100 (V/V) A : B, 2-3 min 0 : 100 (V/V) A : B, 3–3.4 min going from 0 : 100 (V/V) A : B to 100 : 0 (V/V) A : B, and 3.4–5 min 100 : 0 (V/V) A : B. A needle wash solvent of 60 : 40 (V/V) DMSO : ACN was used. The MS operated in ESI^+^ mode with an optimised cone voltage of 50 V, a capillary voltage of 3.0 kV, and a source offset of 60 V. Cone and desolvation gas (N_2_) flows were 180 and 1000 L/h, respectively, while desolvation and source temperatures were set at 500°C and 150°C, respectively. Acquisition was performed in the multiple reaction monitoring (MRM) mode with* m/z* 224.11 to* m/z* 80.84 transition. The collision gas was argon and the applied collision energy was 26 eV. By means of MassLynx® software (V4.1 SCN 843, Waters, Zellik, Belgium), data were acquired and analysed.

## 3. Results

### 3.1. Caco-2 Cell Permeability

**Figure 1:**
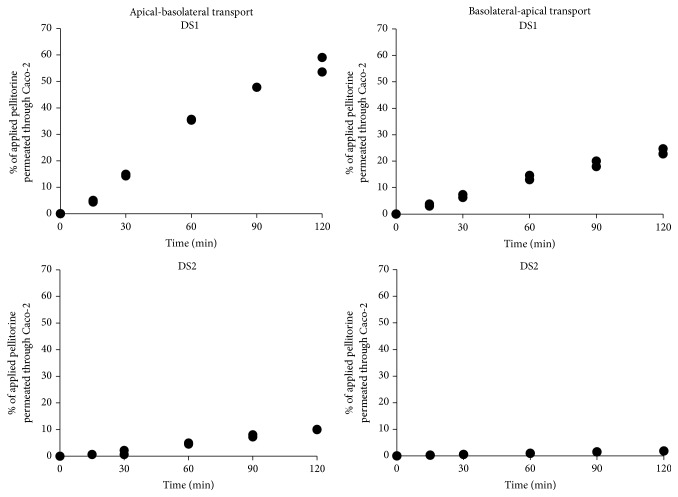
The percentages of pellitorine from the applied dose solutions using dose solution 1 (DS1) and dose solution 2 (DS2) which permeated through the Caco-2 cells monolayer in the course of time. Apical-to-basolateral transport and basolateral-to-apical transport experiments of pellitorine were performed in duplicate (individual results in the same figure).

Our results demonstrated that pellitorine was able to permeate the Caco-2 cell monolayer in both directions: from the apical-to-basolateral side and from the basolateral-to-apical side. The percentages of pellitorine from the applied dose solutions (DS1 and DS2) which permeated through the Caco-2 cells in the course of time are shown in Figure 1. After 120 min, 56.7% (or 0.31 *μ*g pellitorine) of the applied pellitorine with DS1 and 9.98% (or 2.24 *μ*g pellitorine) of the applied pellitorine with DS2 permeated the Caco-2 cell monolayer in the apical-to-basolateral direction. In the basolateral to apical direction, the percentage of pellitorine which permeated through the Caco-2 cells after 120 min is 23.7% (or 0.39 *μ*g pellitorine) with DS1 and 1.84% (or 1.24 *μ*g pellitorine) with DS2. *P*_app,*ab*_ of the positive control propranolol resulted in 19.1 · 10^−6^ cm/s and similar values are documented in literature [32]. The negative control atenolol showed a lower permeability compared to propranolol.

Apparent permeability coefficients of pellitorine of 48.08 ± 0.08 · 10^−6^ cm/s (mean ± SD, *n* = 2) and 5.50 ± 0.06 · 10^−6^ cm/s (mean ± SD, *n* = 2) in case of DS1 and DS2, respectively, were calculated for the apical-to-basolateral transport. For the opposite direction (basolateral-to-apical), *P*_app_ values of pellitorine obtained with DS1 and DS2 are 57.49 ± 6.20 · 10^−6^ cm/s (mean ± SD, *n* = 2) and 2.80 ± 0.09 · 10^−6^ cm/s (mean ± SD, *n* = 2), respectively. Uptake ratios of 0.84 and 1.96 using DS1 and DS2, respectively, are obtained.

### 3.2. Oral Gavage Experiment

**Figure 2:**
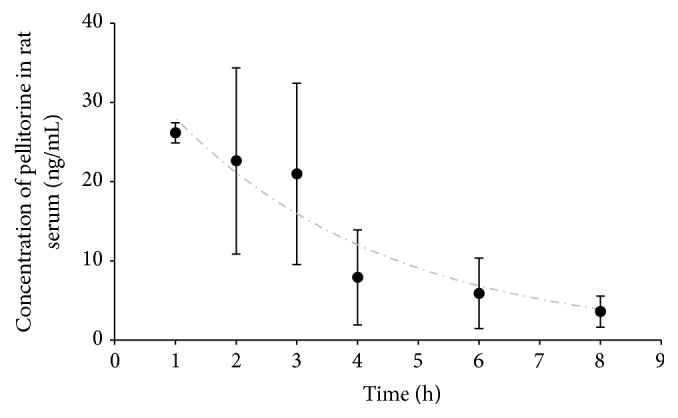
Concentration of pellitorine in rat serum (ng/mL) as a function of time (h) after oral gavage of pellitorine (5 mg pellitorine/kg body weight). Data are fitted according to a one-compartment model (*n* = 2-3, mean, error bars: SEM).

The pellitorine serum concentrations after oral gavage are plotted against time (Figure 2). Pellitorine was able to permeate the intestinal barrier* in vivo*.

The curve was fitted according to a one-compartment model. An elimination rate constant and half-life of 0.28 h^−1^ and 2.46 h were obtained.

### 3.3. Blood-Brain Barrier Transport Kinetics of Pellitorine

**Figure 3:**
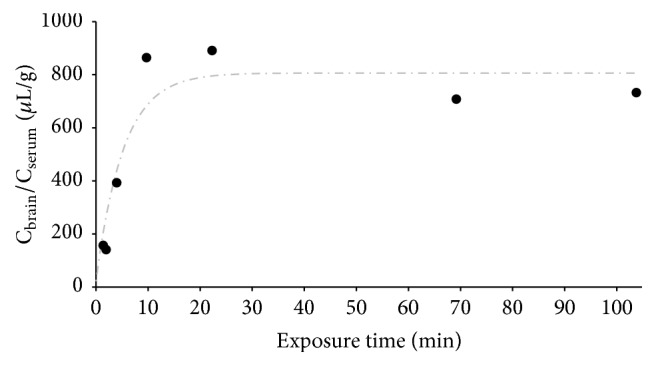
Blood-to-brain transport (multiple time regression experiment) results of pellitorine in mice. The ratio of pellitorine concentration in brain versus serum (*μ*L serum/g brain) is plotted versus the exposure time (min). Start and end point were performed in duplicate. The data were fitted using a biphasic model.

Using the MTR method, pellitorine showed a significant influx into the mouse brain. In Figure 3, the ratio of the concentration of pellitorine in brain and serum is plotted versus the exposure time. The data were fitted using a biphasic model, based on the modified Gjedde-Patlak equation according to Wong et al. [26]. Pellitorine showed a very high and rapid influx into the brains and a *K*_1_ of 153 *μ*L/(g·min) was determined. Moreover, the tissue brain distribution volume (*V*_*g*_) is 792 *μ*L/g. After about 10 min exposure time, a plateau was observed, which is consistent with the efflux of pellitorine out of the brain.

Dermorphin (positive control) clearly showed an influx with a calculated unidirectional influx rate of 0.26 *μ*L/(g·min). The negative control BSA gave a very small, almost negligible brain influx, with a *K*_1_ of 0.12 *μ*L/(g·min). Both controls thus indicated a good performance of the test and are in compliance with those previously reported [29, 33].

The MTR data allowed us to evaluate the elimination kinetics of pellitorine in serum as well. The serum profile of pellitorine followed a two-compartment model. *C*_1_ was 7.02 *μ*g/mL and *C*_2_ was 0.44 *μ*g/mL. The distribution rate constant *α* was 1.56 min^−1^, while the elimination rate constant *β* was 0.15 min^−1^ and an elimination half-life of 4.48 min was obtained.

### 3.4. Capillary Depletion

The fraction of pellitorine that was taken up by the brain parenchyma and the fraction of pellitorine which was trapped in the endothelial cells of the brain capillaries were investigated. 97% of pellitorine (corresponding to 347.3 *μ*L/g) transferred effectively into the brain parenchyma, versus a low percentage of pellitorine remaining in the brain capillaries: 3% (corresponding to 10.0 *μ*L/g). The high absolute values in the brain parenchyma are consistent with the MTR results.

### 3.5. Brain-to-Blood Transport Kinetics of Pellitorine

**Figure 4:**
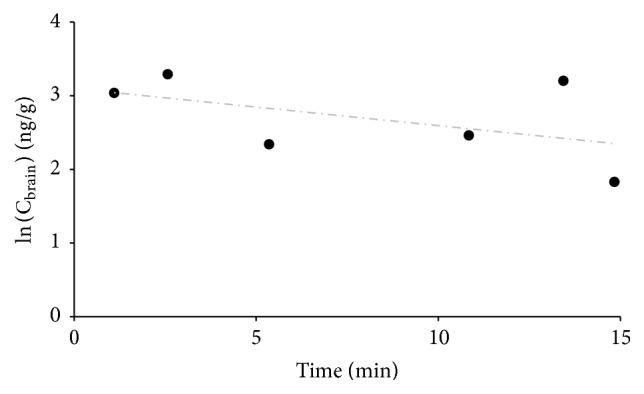
Brain-to-blood transport results of pellitorine in mice. The natural logarithm of the concentration of pellitorine in the brain (ng/g) as a function of the time (min) is given.

A significant efflux of pellitorine out of the brain into the blood was observed after intracerebroventricular injection of the dose solution (Figure 4). The efflux behavior can explain the rapid plateauing observed during the brain influx experiment. The efflux transfer constant *k*_out_ was calculated as 0.05 min^−1^, equal to *t*_1/2,brain_ of 13.8 min.

### 3.6. *In Vitro* Metabolic Stability of Pellitorine

The stability of pellitorine was determined in mouse serum, mouse brain homogenate, and mouse liver homogenate. The results of this study indicate that pellitorine was stable for one hour in serum, in brain as well as in liver (91%–105% recovery).

## 4. Discussion 

To characterize the intestinal absorption of pellitorine, the permeability of pellitorine through Caco-2 cells was investigated using two different dose solutions. The ethanol percentage in both dose solutions was less than 0.5%. The permeation level of pellitorine through Caco-2 cells was higher from the absorptive apical-to-basolateral side (10–57%), compared to the permeation from the basolateral-to-apical side (2–24%). Pellitorine, applied as DS1, better permeated the Caco-2 cells monolayer compared to pellitorine applied as DS2 in both directions, indicating that cosolvents (vitamin E-TPGS, PEG 400, PG) did not influence the permeation level through the cells in a positive way. However, the esterified vitamin E derivative, vitamin E-TPGS, was added to increase the solubility and facilitate the permeation of pellitorine [34, 35]. The *P*_app_ values obtained for pellitorine (0.3 · 10^−5^ to 5.8 · 10^−5^ cm/s) were all above 1 · 10^−6^ cm/s, suggesting an almost complete intestinal absorption, and are similar to values reported by Matthias et al. [36] of the mono- and diene-NAAs from* Echinacea* plant species. Given the physicochemical properties of pellitorine (log⁡*P* = 3.65, MW = 223.36 g/mole), transcellular passive diffusion seems favourable for pellitorine [37]. This was confirmed in our study, as for both dose solutions containing pellitorine, the ratio of *P*_app_ values between the apical-to-basolateral and the basolateral-to-apical direction or vice versa was ≤ 2, indicating that no active transport was involved [38]. As the efflux ratios were not greater than two, there is no efflux of pellitorine. Next to this* in vitro *evaluation of the intestinal barrier properties of pellitorine, a confirmatory* in vivo* oral gavage experimentin rats was performed. A liquid dose solution of pellitorine also containing EtOH, PG, Vit E-TPGS, and PEG 400 was orally administered to rats. Pellitorine diffused through the gut barrier and was observable in rat serum. These results confirm the outcome of the initial* in vitro* Caco-2 cell permeability study. The elimination half-life of pellitorine was 2.46 h, which is in the same range as the elimination half-life of 1.20 h, obtained by Woelkart et al. [39], who investigated the plasma concentration of dodeca-2E,4E,8E,10E/Z-tetraenoic acid isobutylamides from* Echinacea* via oral gavage in rats.

Once in the blood, pellitorine can distribute to extravascular compartments in order to exert a biological function. In the present study, the blood-brain barrier transport characteristics of pellitorine were explored in an* in vivo* mice experiment. Pellitorine was intravenously injected to the mice in a solution containing EtOH and dimethylacetamide, which are both frequently used as cosolvents in injectable pharmaceutical formulations, and tween 80, which is a traditional surfactant, and diluted in lactated Ringer's solution containing BSA [40, 41]. In our study, no toxicity was observed when performing the experiment and it is likely that the solvents did not have an influence on the BBB integrity, as lower doses are used than doses reported in literature which can cause blood-brain barrier disruption. A dose of tween 80 up to 30 mg/kg and 1–4 g/kg ethanol can disrupt the BBB, while in this study only 3 mg/kg tween 80 and 0.04 g EtOH/kg body weight were administered [42]. In the current study, a high initial unidirectional influx rate of 153 *μ*L/(g·min) was observed, indicating that pellitorine rapidly penetrated the BBB after administration, which is consistent with the lipophilicity of pellitorine. In addition, pellitorine was distributed in the brains as follows: 97% in the parenchyma and only 3% in the capillaries, making it plausible to exert CNS effects. Badhe et al. [43] already showed antidepressant activity of a hydroalcoholic* Anacyclus pyrethrum* root extract (50, 100, and 200 mg/kg, p.o.) in albino mice. Another study demonstrated the anticonvulsant and myorelaxation activity of an ethanolic AP root extract in albino mice. A dose dependent effect was observed against maximum electroshock test and rotarod test after intraperitoneal administration of the extract in doses of 200, 400, and 600 mg/kg [44]. Sujith et al. [14] evaluated neuropharmacological activity of an ethanolic AP root extract (50, 100, and 200 mg/kg, p.o.) in albino Wistar rats. Nootropic activity was observed in rats in a dose dependent way using the elevated plus maze (EPM) test. Furthermore, the extract also showed antidepressant activity in a forced swim test. Another study showed the anticonvulsant activity against pentylenetetrazole (PTZ) of a hydroalcoholic root extract of AP after oral administration of 50, 100, 250, and 500 mg/kg to Wistar rats. The observed effect was dose dependent. In addition, protection against MES induced seizures was observed in a dose dependent way after administration of the extract in doses of 250, 500, and 1000 mg/kg. Furthermore, the extract prevented seizure induced oxidative stress and showed protective effects against cognitive impairment in rats in a dose dependent manner [45]. Sujith et al. [46] showed a dose dependent cognitive improvement of an ethanolic root extract of AP (50, 100, and 200 mg/kg) after oral administration to albino Wistar rats in an EPM test and passive avoidance paradigms. Moreover, the extract enhanced short-term social memory in a social recognition task. Zaidi et al. [47] showed anticonvulsant activity against PTZ in a dose dependent manner in mice after administration of a chloroform AP root extract in a concentration of 100, 200, 400, and 800 mg/kg. Also, against bicuculline, the 800 mg/kg AP extract showed anticonvulsant activity. Furthermore, anxiolytic behavior was also observed in an EPM model after administration of 800 mg/kg AP extract. Administration of 1600 mg/kg AP extract showed impaired motor coordination in a rotarod test. Another study showed that pretreatment of albino mice with 100, 250, and 500 mg/kg (p.o.) hydroalcoholic root AP extract increased myoclonic jerk latency and delay in the development of kindling. Protection against memory deficit was observed after pretreatment of the AP extract in doses of 100, 250, and 500 mg/kg (p.o.) in PTZ kindled mice by decreasing oxidative stress and ROCK II expression [48]. Other research demonstrated the anticonvulsant activity of an ethanolic root extract of* Anacyclus pyrethrum* (200, 400 mg/kg, p.o.) in a MES model in albino mice. 400 mg/kg AP extract was protective against PTZ model [13].

In the previously described effect studies, the AP extract was administered to rats or mice in doses ranging between 50 and 1600 mg extract/kg body weight. Assuming that the concentration of pellitorine is 1.55% w/w (determined on the ethanolic AP extract used in this study), the pellitorine dose ranges between 0.8 and 24 mg/kg. Since our study showed a maximum serum concentration of 26 ng/mL pellitorine after oral administration of 5 mg pellitorine/kg to rats (Figure 2) and assuming pellitorine follows a linear kinetic, the maximum serum concentration of pellitorine in the previously described effect studies is between 4.0 and 129.0 ng/mL. Our BBB data (Figure 3) showed a brain/serum concentration ratio of 792 *μ*L/g at equilibrium, so that the brain concentrations of pellitorine in the previous effect studies correspond to 3.2 to 102.1 ng pellitorine/g brain. Looking at compounds used for the treatment of CNS diseases (e.g., multiple sclerosis, experimental autoimmune encephalomyelitis, and epilepsy), administered at efficacious doses, drug concentrations in the brain ranging between 15 and 620 ng/g brain were found [49–51]. Hence, our BBB study corroborates well with the previously described effect studies using the AP extract and the brain concentrations of other CNS-active drugs.

## 5. Conclusion

In the current study, the intestinal barrier properties of pellitorine were investigated* in vitro* and* in vivo*. It was shown that pellitorine was able to cross the Caco-2 cell monolayer and the rat gut after oral administration. Furthermore, it was demonstrated that, after intravenously administration of pellitorine to mice, pellitorine significantly and rapidly penetrated the BBB reaching the brain parenchyma. The biphasic course of the influx can partly be explained by an observed significant efflux of pellitorine out of the brain. These pharmacokinetic findings support the observed activities using the* Anacyclus pyrethrum* extract in the treatment of CNS diseases.
